# Efficacy of Local and Whole‐Body Phototherapy for the Treatment of Various Types of Alopecia Areata

**DOI:** 10.1111/phpp.70061

**Published:** 2025-12-03

**Authors:** Aya Yamamoto, Yuki Enomoto, Mai Sakurai, Oki Watanabe, Takashi Sakaida, Yoshifumi Kanayama, Akimichi Morita

**Affiliations:** ^1^ Department of Geriatric and Environmental Dermatology Nagoya City University Graduate School of Medical Sciences Nagoya Japan

**Keywords:** alopecia areata, phototherapy, SALT score

## Abstract

**Background/Purpose:**

Alopecia areata (AA) is a non‐scarring autoimmune hair loss disorder. Although phototherapy has been utilized to treat AA, its efficacy and mechanisms remain unclear. This study aimed to evaluate the effectiveness of phototherapy in AA and determine whether treatment outcomes vary depending on patient characteristics.

**Methods:**

We retrospectively reviewed 49 patients with various types of AA who received at least 10 sessions of either local or whole‐body phototherapy. The severity of hair loss was assessed using the Severity of Alopecia Tool (SALT) score. Patient demographics, disease type, clinical severity, and laboratory data were analyzed in relation to treatment outcomes.

**Results:**

Of the 49 patients, 32 received local phototherapy and 17 received whole‐body irradiation. There was no significant difference in the SALT improvement rate between local and whole‐body irradiation. Significant factors contributing to treatment response included first‐onset AA (*p* = 0.03), treatment initiation within 1 year of onset (*p* = 0.0069), and age over 40 (*p* = 0.0027). Patients with multiple or totalis types of AA demonstrated better responses compared to universalis or ophiasis. Hair regrowth was typically observed after 20–30 treatment sessions.

**Conclusion:**

Phototherapy is an effective treatment for certain subtypes of AA, especially when initiated early after disease onset. Local irradiation showed comparable effectiveness to whole‐body irradiation. These findings support the early initiation of phototherapy as a therapeutic strategy for AA, particularly in older patients and those with limited disease duration.

## 1 Introduction

Alopecia areata (AA) is a common hair loss condition that does not result in scarring. Approximately 150 million people worldwide are estimated to be affected, with a lifetime risk of 2% [1]. Five different types of AA are known: single, multiple, totalis, ophiasis, and universalis [2]. While 67% of patients with the single form of AA will experience spontaneous resolution within a year of onset, severe forms such as alopecia totalis and universalis result in the loss of large areas of hair and can be difficult to treat [2].

AA is a type of autoimmune disease in which a targeted autoimmune response against growing hair follicles leads to a breakdown of immunologic privilege, resulting in hair destruction and hair loss [3]. Disruption of the immune system in the hair follicles is thought to trigger an autoimmune response, in which certain types of T cells primarily target autoantigens in the hair follicles [3]. The attack on the hair follicles induces inflammation, and recent studies suggest that type 2 and Th17‐related mediators play a role in its development [4]. Levels of Th17‐related cytokines, including IL‐17 and IL‐22, significantly increase in individuals with AA [5]. An imbalance between Th17 cells and Tregs is implicated in the pathogenesis of various immune‐mediated diseases [6, 7]. In psoriasis, Tregs in the peripheral blood are dysfunctional, and phototherapy restores Treg function in the peripheral blood [8]. Treg function is also impaired in the peripheral blood of patients with AA [9].

We hypothesized that whole‐body irradiation, rather than local irradiation, would stimulate hair growth in patients with refractory AA. Therefore, we compared the efficacies of whole‐body irradiation and local irradiation in phototherapy for various types of AA.

## 2 Materials and Methods

### 2.1 Participants and Phototherapy Method

Forty‐nine patients undergoing phototherapy for AA (universalis: 31, multiple: 11, totalis: 4, ophiasis: 3) at Nagoya City University Hospital during a 2‐year period (from January 2011 to October 2013) and who received at least 10 irradiation treatments were included. The study was conducted following protocols approved by the Ethics Review Board of Nagoya City University, in accordance with the tenets of the Declaration of Helsinki and the Ethical Guidelines for Clinical Research (#60‐23‐0084).

Study participants were evaluated based on the Severity of Alopecia Tool (SALT) score after every 10 irradiation sessions. Four irradiation devices were used: the whole‐body irradiation devices were the UV7002 (Waldmann, Schwenningen, Germany) and Dermaray 800 (Toshiba Medical Supply Co Ltd., Tokyo, Japan), and the local irradiation devices were the Dermaray 200 (Toshiba Medical Supply Co Ltd., Tokyo, Japan) and Therabeam Slim (USHIO, Tokyo, Japan). Both the type of irradiation and equipment used were randomly selected for each patient. Irradiation protocols are shown in Table 1. The UV7002 was used to irradiate the entire body in the standing position, and the Dermaray 800 was used to irradiate the entire body from the front and back. The Dermaray 200 was used to irradiate the front and back of only the head, and the Therabeam Slim was used to irradiate only the head from the front, back, left, and right.

**TABLE 1:** Patient background by disease type.

	Whole	Universalis	Totalis	Multiple	Ophiasis
Patient	49	31	4	11	3
Men (%)	40.8	54.8	0	27.3	0
Age (years [SD])	37.7 (10.9)	35.6 (10.0)	46.8 (6.7)	42.5 (13.6)	38.0 (0.0)
Duration (years [SD])	7.86 (8.86)	8.71 (8.86)	2.75 (2.36)	5.0 (6.6)	16.33 (16.29)
Incipient (%)	57.1	58.1	75	63.6	0
Medical history of AD (%)	38.8	48.4	0	27.3	33.3
SALT score at the start (SD)	84.42 (24.15)	93.66 (14.37)	93.55 (9.65)	69.61 (24.24)	31.0 (28.93)

The devices used for phototherapy emitted UVB light at wavelengths of either 311 ± 2 nm (narrowband UVB) or 308 nm (excimer light). The starting dose for narrowband UVB was 0.3 J/cm^2^, increased by 20% every session, up to a maximum of 2.0 J/cm^2^. For excimer light, the initial dose was 150 mJ, increased by 20% per session. Cumulative doses varied depending on treatment duration and tolerance but typically ranged from 4.7 to 110.0 J/cm^2^ for narrowband UVB and 3.7 to 51.7 J/cm^2^ for excimer light over the course of therapy.

During the study period, no systemic immunosuppressive agents (e.g., systemic corticosteroids, JAK inhibitors) were permitted. A small number of patients continued taking oral cepharanthine; however, their use was not systematically recorded or analyzed. Therefore, all results should be interpreted primarily as monotherapy effects of phototherapy.

### 2.2 Clinical Data

The data collected for this study included the patient's age, sex, disease duration, laboratory data, and history of smoking and alcohol use. Information regarding smoking and alcohol use was obtained through self‐reported questionnaires.

### 2.3 Statistical Analysis

A nonparametric Mann–Whitney *U* test was applied for comparisons between two independent datasets, while comparisons among multiple groups were evaluated using the Steel‐Dwass test. The analysis was conducted using GraphPad Prism software developed by the GraphPad Software company based in San Diego, California.

## 3 Results

### 3.1 Participant Characteristics

The 49 patients comprised 20 men and 29 women with a mean (SD) age of 37.7 (10.9) years and a mean (SD) disease duration of 7.9 (8.9) years. Among the patients, 57.1% had AA for the first time, and 38.8% had concomitant atopic dermatitis. At the beginning of the study, the mean (SD) SALT score was 84.4 (24.2). The disease types were represented as follows: universalis, 31; totalis, 4; multiple, 11; and ophiasis, 3.

Among patients with the universalis type, the male:female ratio was 17:14, the mean age was 35.6 (10.0) years, the mean disease duration was 8.7 (8.9) years, and 18 patients (54.8%) were first‐episode cases. Of the 31 patients with the universalis type, 15 (48.4%) had a history of atopic dermatitis, and the mean SALT score at the start of treatment was 93.7 (14.4).

The four patients with the totalis type were female, with a mean age of 46.8 (6.7) years, a mean disease duration of 2.8 (2.4) years, and 3 of the 4 patients (75%) were first‐onset cases. None of the patients had a history of atopic dermatitis, and the mean SALT score at the start of treatment was 93.6 (9.7).

Among the 11 patients with the multiple type, the male‐to‐female ratio was 3:8, the mean age was 42.5 (13.6) years, the mean disease duration was 5.0 (6.6) years, and 7 patients (63.6%) were first‐onset patients. Three patients (27.3%) had a history of atopic dermatitis, and the mean SALT score at the start of treatment was 69.61 (24.2).

All 3 patients with the ophiasis type were female, with a mean age of 38.0 (0.0) years and a mean disease duration of 16.3 (16.3) years; none of the three patients was first‐episode cases. One patient (33.3%) had a history of atopic dermatitis, and the mean SALT score at the beginning of treatment was 31.0 (28.9) (Table 1).

The severity of hair loss was assessed using the SALT score, which categorized cases into five levels: S1 (< 25% total hair loss), S2 (25%–49% hair loss), S3 (50%–74% hair loss), S4 (75%–99% hair loss), and S5 (100% hair loss). Among the 49 patients, 3 were classified as S1 with a hair loss area of 0%–24%. All S1 patients were female, with a mean age of 47 (15.6) years and a mean disease duration of 5.67 (3.06) years. One of the patients (33.3%) was experiencing a first episode of AA and one patient (33.3%) had a history of atopic dermatitis. The mean SALT score at the beginning of treatment was 16.3 (3.4).

In the S2 category, there were two patients (1 male, 1 female) with a mean age of 43.5 (3.5) years and a mean disease duration of 3.5 (3.54) years. Both patients had previous episodes of AA, and both had a history of atopic dermatitis. The mean SALT score at the beginning of treatment was 39.9 (3.25).

In the S3 category, there were six patients (1 male, 5 females) with a mean age of 35 (7.1) years and a mean disease duration of 9.0 (13.2) years. Of the 6 patients, 3 (50%) were experiencing their first episode of AA, and 2 (33.3%) had a history of atopic dermatitis. The mean SALT score at the beginning of treatment was 62.6 (7.0).

For the S4 category, there were 15 (8 males, 7 females) patients with a mean age of 41 (7.1) years and a mean disease duration of 9 (6.5) years. Of the 15 patients, 11 (73.3%) were first‐onset cases and 1 (33.3%) had a history of atopic dermatitis. The mean SALT score at the beginning of treatment was 88.82 (8.8). Finally, the S5 category included 23 patients with S5 (10 males, 13 females) with a mean age of 35.6 (12.8) years and a mean disease duration of 9.8 (9.7) years. Thirteen patients (56.5%) in this group were first‐onset cases and 9 (39.1%) had a history of atopic dermatitis. The mean SALT score at the beginning of treatment was 100 (0) (Table 2). Overall, 77.5% of the patients experienced severe hair loss, with an affected area ≥ 75%, categorized as S4 and S5.

**TABLE 2:** Patient background by area of hair loss.

	Whole	S1	S2	S3	S4	S5
Patient	49	3	2	6	15	23
Men (%)	40.8	0	50	16.7	53.3	43.5
Age (years [SD])	37.7 (10.9)	47 (15.6)	43.5 (3.5)	35 (7.1)	41 (7.1)	35.6 (12.8)
Duration (years [SD])	7.86 (8.86)	5.67 (3.06)	3.5 (3.54)	9.0 (13.16)	9 (6.52)	9.78 (9.65)
Incipient (%)	57.1	33.3	0	50	73.3	56.5
Medical history of AD (%)	38.8	33.3	100	33.3	33.3	39.1
SALT score at the start (SD)	84.42 (24.15)	16.27 (3.41)	39.9 (3.25)	62.58 (6.97)	88.82 (8.76)	100 (0)

### 3.2 Improvement Rate by Patient Background

The study analyzed the rate of improvement in the SALT score according to the patient background. The SALT improvement rate did not significantly differ between male and female patients (*p* = 0.6681). Patients with a disease duration < 1 year had a significantly faster SALT improvement rate than those with a disease duration ≥ 1 year (*p* = 0.0008).

Patients who were ≥ 40 years of age exhibited a significantly improved SALT rate (*p* = 0.0340). The rate of improvement did not significantly differ between patients with a body mass index (BMI) ≥ 25 or between patients with a BMI < 25 (*p* = 0.2147). No significant difference in the rate of SALT improvement was detected between those with and without concomitant atopic dermatitis (*p* = 0.3170). In addition, we found no association between the rate of improvement and smoking habit (*p* = 0.4760) or alcohol consumption (*p* = 0.6162; Table 3).

**TABLE 3:** SALT score improvement rate according to patient background.

	Patients, *n* (%)	SALT improvement rate, Mean (SD)	*p*
Sex			
Female	29 (59.2)	31.57 (39.68)	
Male	20 (40.8)	32.12 (39.03)	
			0.6681
Duration of illness			
< 1 year	12 (24.5)	67.19 (40.04)	
≧ 1 year	37 (75.5)	20.31 (31.40)	
			0.0008
BMI			
< 25	41 (83.7)	34.59 (39.95)	
≧ 25	8 (16.3)	17.45 (32.15)	
			0.2147
Medical history of atopic dermatitis			
AD (+)	19 (38.8)	35.47 (42.58)	
AD (−)	30 (61.2)	25.98 (32.85)	
			0.3170
Smoking			
Smoking	10 (20.4)	33.65 (40.95)	
No smoking	39 (79.6)	31.32 (39.04)	
			0.4760
Alcohol drinking habit			
Drinking habit	20 (40.8)	34.18 (37.19)	
No drinking habit	27 (55.1)	29.96 (41.38)	
Unknown	2 (4.1)	32.70 (31.90)	
			0.6162^a^

^a^ Forty‐seven people were considered, excluding the unknowns.

### 3.3 Improvement Rate Based on AA Type

The overall remission rate, as measured by SALT100, was 8.2%, including a 50% remission rate in cases with AA totalis, 18% in multiple cases, and 0% in other AA types. For SALT90, the achievement rates were 75% overall, 36% in multiple cases, and 3.2% in universalis cases. SALT75 was achieved in 75% of all cases, 46% in multiple cases, and 3.2% in universalis cases. The SALT50 target was reached by 35% of all patients, including 64% of multiple cases, 23% in universalis cases, and 75% of totalis cases.

**FIGURE 1:**
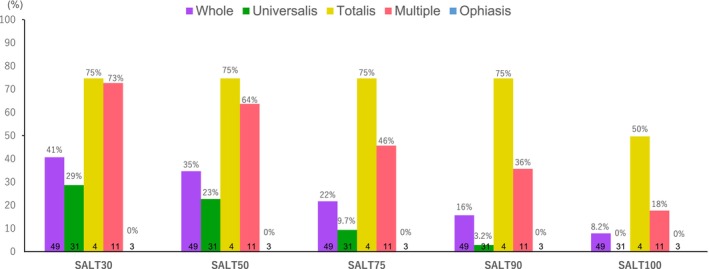
Improvement rates by AA type. The rate of improvement for each disease type is shown. The improvement rate was high for the totalis and multiple AA types.

**FIGURE 2:**
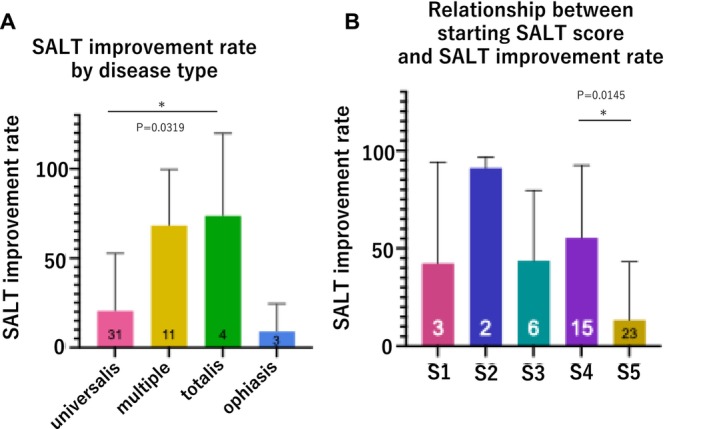
(A) Comparison of improvement rates between disease types. Significant differences in improvement rates were detected between the totalis and universalis AA types. (B) Improvement rate according to severity (area of hair loss at treatment initiation). The severity of hair loss at the beginning of irradiation and the improvement rate were examined. A significant difference in the improvement rate was detected between the S4 (75%–99% hair loss area) and S5 (100% hair loss area) severity levels.

The SALT30 rate was 75% in totalis cases, 73% in multiple cases, 29% in universalis cases, and 0% in ophiasis cases (Figure 1). The mean (SD) improvement in the SALT score across all patients was 34.8 (39.4). By disease type, the improvement rates were 20.5 (32.3) for universalis cases, 68.03 (31.5) for multiple cases, 73.68 (46.3) for totalis cases, and 9.03 (15.7) for ophiasis cases. A significant difference in the improvement rates was detected between totalis and universalis cases (*p* = 0.0319, Dunn's multiple comparison test; Figure 2A). The inclusion of acute diffuse and total alopecia of the female scalp within the totalis classification led to a favorable improvement rate, suggesting a positive prognosis for this subgroup.

### 3.4 Improvement Rate by Severity (Hair Loss Area at Start)

The severity of hair loss at the start was classified as S1 (> 25% of total hair loss area), S2 (25%–49% of hair loss area), S3 (50%–74% of hair loss area), S4 (75%–99% of hair loss area), and S5 (100% of hair loss area), and the improvement rates differed significantly between S4 and S5 (*p* = 0.0145, Dunn's multiple comparison test). We believe that the good improvement rate of S4 was due to the inclusion of the aforementioned acute diffuse and total alopecia of the female scalp in the S4 category (Figure 2B).

### 3.5 Improvement Rate Between Cases With Whole‐Body Irradiation and Local Irradiation

Among the 49 patients with alopecia areata, 32 received local irradiation (16 with universalis, 3 with totalis, 10 with multiple, and 3 with ophiasis) and 17 received whole‐body irradiation (15 with universalis, 1 with totalis, and 1 with multiple). Patients who completed treatment with improvement were considered “clinical resolution.” For patients with > 1% improvement, treatment was considered “effective,” and for patients who showed no improvement, treatment was considered “ineffective.”

**FIGURE 3:**
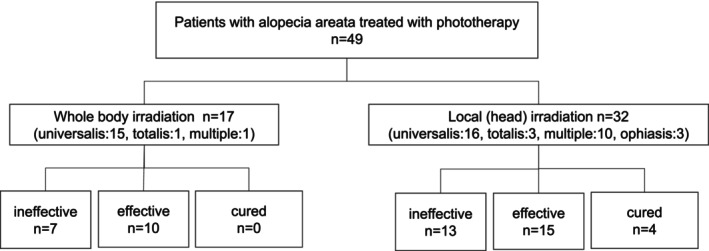
Patients who received local or whole‐body irradiation. Of the 49 patients with AA, 17 received whole‐body irradiation and 32 received local irradiation. Among those receiving whole‐body irradiation, none showed clinical resolution; treatment was effective in 10 cases and ineffective in 7. Among those receiving local irradiation, 4 showed clinical resolution, and treatment was effective in 15 and ineffective in 13.

Among the patients who received local irradiation, 4 achieved clinical resolution and ended the treatment, 15 showed improvement, and 13 did not respond to the treatment. Of the patients who received whole‐body irradiation, 0 showed clinical resolution and completed the treatment, 10 showed improvement, and 7 did not respond to treatment (Figure 3).

**FIGURE 4:**
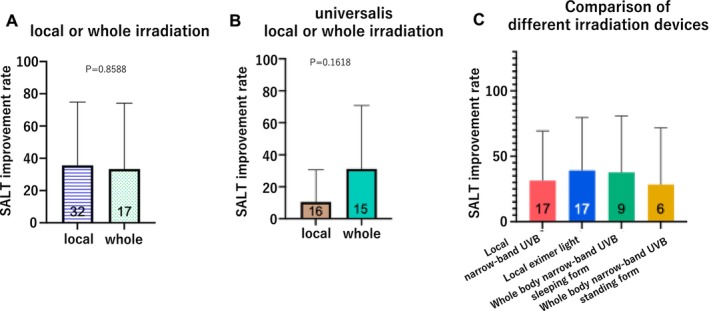
(A) SALT improvement rates compared between whole‐body and local irradiation. No difference in the SALT improvement rate was detected between patients receiving whole‐body irradiation and those receiving local irradiation. (B) Comparison of whole body irradiation and local irradiation in patients with universalis AA. A comparison of the effectiveness of whole‐body irradiation and local irradiation against the most severe form of the disease, the universalis type, showed no significant difference in the rate of improvement. (C) Comparison of irradiation devices. The rate of improvement by irradiation device was examined, and no significant difference was detected.

The rate of improvement in the SALT score was compared between the 32 patients who received local irradiation and the 17 patients who received whole‐body irradiation; no significant difference was detected between groups (*p* = 0.8588; Figure 4A). The SALT improvement rate was compared between patients with whole‐body irradiation and those with local irradiation among the 31 patients with universalis, the most severe form of the disease, and no significant difference was detected (*p* = 0.1618; Figure 4B). Furthermore, no significant difference in the rate of SALT improvement was detected among the four types of irradiation devices used (*p* = 0.6991; Figure 4C).

### 3.6 Laboratory Data

**FIGURE 5:**
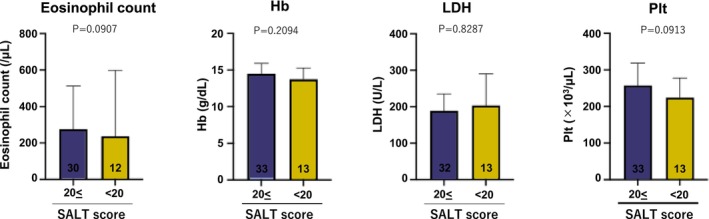
Comparison of clinical data at the start. Clinical data at the time of treatment initiation were compared between patients with a SALT score ≥ 20 at the end of irradiation and patients with a SALT score ≤ 20. No significant differences in the eosinophil counts, hemoglobin (Hb), lactate dehydrogenase (LDH), or platelet (Plt) counts were detected between groups.

The clinical data (eosinophil count [/μL], hemoglobin [g/dL], lactate dehydrogenase [U/L], and platelet count [×10^3^/μL]) of 45 patients for whom clinical data were available at the first visit were compared between patients who achieved a SALT score ≤ 20 at the end of treatment and those who had not. The eosinophil counts of 42 of these patients were analyzed. No significant differences in the eosinophil count were detected (*p* = 0.0907, Figure 5), hemoglobin (*p* = 0.2094, Figure 5), lactate dehydrogenase (*p* = 0.8287, Figure 5), or platelet count (*p* = 0.0913, Figure 5).

### 3.7 Irradiation Times

**FIGURE 6:**
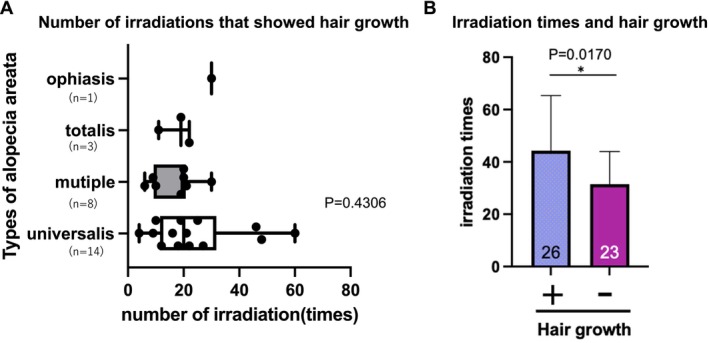
(A) Number of irradiations leading to hair growth by AA type. The number of irradiations needed to see hair regrowth in each disease type was plotted. The mean number of irradiations needed to see hair regrowth among all 49 patients was 21, with no difference between AA types. The totalis and ophiasis types were omitted from the statistical analysis due to their small numbers, and only universalis and multiple were statistically evaluated. (B) Number of irradiations and hair growth. The number of irradiation times was higher in the group that showed hair growth. Irradiation was continued in the group for which it was effective and discontinued for those who did not respond.

Of the 49 patients, 26 (53.1%) showed hair regrowth following phototherapy. Hair regrowth was observed in 14 patients with the universalis type, eight with the multiple type, three with the totalis type, and one with the ophiasis type. Hair growth was observed after a mean of 21.3 (13.2) irradiation times. The number of irradiations required for hair regrowth did not differ significantly among the different AA types (Figure 6A). The totalis and ophiasis types were omitted due to their small numbers, and the universalis and multiple types were examined. Comparison of the number of irradiations between groups with and without hair growth revealed that the group with hair growth received a significantly higher number of irradiations (*p* = 0.0170, Figure 6B). After 10 sessions, the irradiation treatments were stopped if not effective and continued if effective.

No serious adverse events were observed during the study period. Mild erythema was reported in three patients and transient pruritus in four patients, all of which resolved without intervention. No blistering or long‐term adverse effects were noted.

## 4 Discussion

Phototherapy for AA is more effective for cases with the multiple and totalis types. The improvement rate in the totalis type may have been better because this classification included acute diffuse and total alopecia of the female scalp, a type with a better prognosis [10]. No difference was detected between whole‐body irradiation and local irradiation for either of the irradiation devices used.

Patients who began irradiation treatments within 1 year of disease onset exhibited higher improvement rates, suggesting that phototherapy should begin as early as possible after disease onset. Younger patients (< 40 years of age) were more refractory to phototherapy, and the improvement rate was poor. In patients whose hair regrew, the mean (SD) number of irradiation sessions required was 21.3 (13.2), and no significant difference was detected between disease types. In general, phototherapy was effective for AA after approximately 20–30 sessions.

The hair follicles are characterized by immunologic privilege, a state in which the follicle does not produce an autoimmune response to self‐antigens expressed within the follicle. In other words, the expression of MHC class 1 is decreased and the secretion of transforming growth factor beta 1 (TGFβ1) is increased [11]. AA is a type of autoimmune disease in which an autoimmune reaction targeting the growing hair follicle destroys its immunologic privilege, resulting in hair destruction and alopecia. The destruction of the immune privilege of the hair follicle is thought to trigger an autoimmune reaction in which autoreactive cytotoxic CD8^+^NKG2D^+^ T cells primarily target exposed hair follicle autoantigens [12]. Originally, the Th1 axis was thought to play an important role, but recent data indicate the involvement of the Th2 and Th17 axes [4, 5]. Imbalances between Th17 cells and Tregs are implicated in the etiology of various immune‐mediated diseases [6, 7]. Tregs are thought to play an important role in suppressing autoimmune diseases by attenuating the response of CD8^+^ cells [13]. TGFβ1 levels in the peripheral blood are decreased within 1 year of onset in patients with active AA [14], suggesting that TGFβ1 is mainly secreted by Tregs and that Tregs are decreased in patients with active AA. In this study, we did not analyze the mechanisms underlying the effects of phototherapy.

Ultraviolet (UV) radiation affects the cells responsible for immunity and influences immune responses. UV irradiation causes local immunosuppression and systemic immunosuppression, and Tregs are thought to be active in local immunosuppression. Shreedhar et al. [15] suggested that UV light induces regulatory T cells, which may prevent the activation of Th1 cell‐mediated immune responses. Furuhashi et al. [8] showed that phototherapy restores dysfunctional Tregs in patients with psoriasis and resolves the Th17/Treg imbalance. In addition, Tregs in the skin preferentially localize to the perifollicular area [16]. Considering that the effects of whole‐body irradiation and local irradiation were not different in the present study, it is possible that Tregs induced around hair follicles in the head by UV irradiation help improve the pathogenesis of AA.

Phototherapy is the most effective treatment for AA and should be started as soon as possible after the onset of the disease. Comparable effects are produced by various types of UV irradiation, including narrowband UVB at 311 ± 2 nm or excimer light at 308 nm. Therefore, treatment should commence with the available equipment as soon as possible. At least 20–30 irradiations are necessary before evaluating the effectiveness. In the previous studies, PUVA therapy for alopecia areata showed varying response rates. Mitchell et al. reported that 36% of patients achieved ≥ 75% hair regrowth [17], while Mohamed et al. found that 85% of patients showed good clinical outcomes [18]. In contrast, reports on narrowband UVB (NB‐UVB) are limited. For example, Jury et al., who studied pediatric cases, noted that 83% of patients showed no improvement [19].

This study demonstrates that phototherapy is a viable treatment option for alopecia areata, particularly in patients with the multiple and totalis subtypes. No significant difference in efficacy was observed between whole‐body and local irradiation. Early initiation of treatment—especially within 1 year of disease onset—was associated with better outcomes, and patients over the age of 40 responded more favorably to phototherapy. Approximately 20–30 irradiation sessions were needed to observe hair regrowth. Although long‐term follow‐up was limited, these findings highlight the importance of early intervention and support the continued use of phototherapy in the clinical management of alopecia areata. Future studies should investigate recurrence rates and predictors of sustained remission.

## Author Contributions

**Aya Yamamoto:** conceptualization; data curation; formal analysis; investigation; visualization; writing – review and editing. **Yuki Enomoto:** investigation; writing – review and editing. **Yoshifumi Kanayama:** investigation; writing – review and editing. **Mai Sakurai:** investigation; writing – review and editing. **Oki Watanabe:** investigation; writing – review and editing. **Takashi Sakaida:** investigation; writing – review and editing. **Akimichi Morita:** conceptualization; funding acquisition; project administration; supervision; writing – review and editing.

## Conflicts of Interest

The authors declare no conflicts of interest.

## Data Availability

The data that support the findings of this study are available on request from the corresponding author. The data are not publicly available due to privacy or ethical restrictions.
